# P-1107. Evaluation of Candidemia and Antifungal Use in ECMO Patients at a Large Volume ECMO Center

**DOI:** 10.1093/ofid/ofaf695.1302

**Published:** 2026-01-11

**Authors:** Madison D Granger, Rongbing Xie, Owen Albin, Seth Edwards, Joshua Stripling, Todd P McCarty, Rachael A Lee, Jeremey Walker

**Affiliations:** University of Alabama at Birmingham Heersink School of Medicine, Birmingham, AL; UAB Medical Center, Birmingham, Alabama; University of Michigan Medical School, Ann Arbor, MI; UAB Hospital, Birmingham, Alabama; University of Alabama at Birmingham, Birmingham, Alabama; University of Alabama at Birmingham, Birmingham, Alabama; University of Alabama at Birmingham, Birmingham, Alabama; University of Alabama Birmingham, Birmingham, Alabama

## Abstract

**Background:**

*Candida* species are a common cause of bloodstream infections in patients receiving Extracorporeal Membrane Oxygenation (ECMO) therapy and a driver of morbidity and mortality. [1, 2]. Risk for candidemia is higher with VV ECMO and duration on ECMO therapy >3 weeks [3]. There is little data describing antifungal use in ECMO or on the use of rapid diagnostic testing with T2Candida (T2C) to identify candidemia in these patients.

**Figure:**
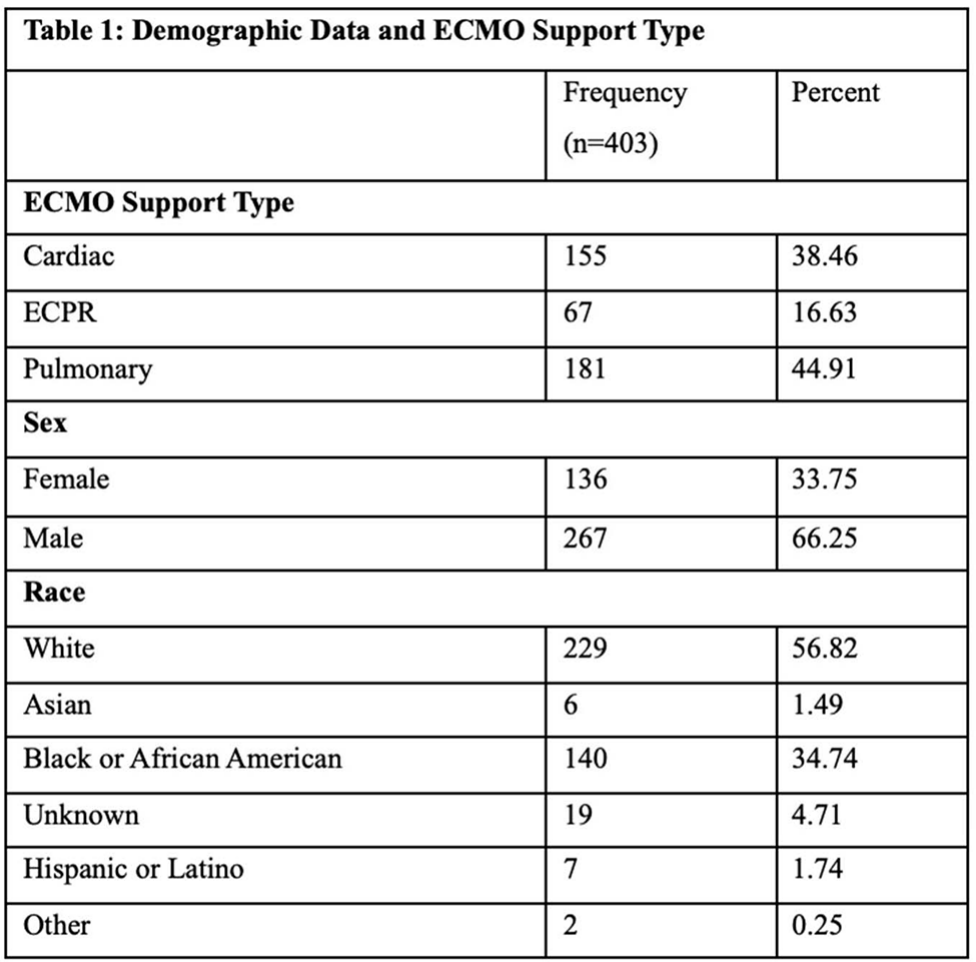

**Figure:**
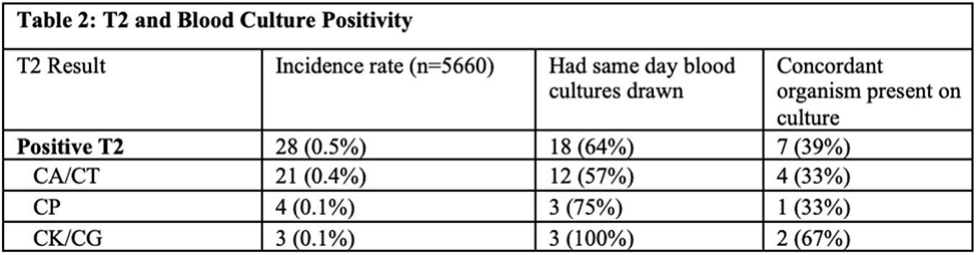

**Methods:**

We conducted a retrospective cohort study of adult patients requiring ECMO from 1/1/2021-12/31/2023. Only the first 30 days of ECMO and first ECMO run was included in the analysis. Candidemia was defined as identification of *Candida* species in at least 1 culture bottle. Antifungal use was documented over the duration of their ECMO run, or up to 30 days.

**Figure:**
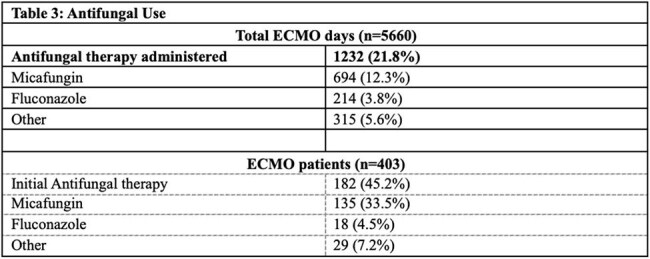

**Results:**

A total of 403 ECMO patients and 5,660 ECMO days were included. Patient demographics are shown in Table 1. Of the 403 patients, 48 (11.9%) had a positive T2C result or blood culture for candida isolate and 29 (6%) had blood culture proven candidemia. T2C testing was performed in 33% of confirmed candidemia cases and showed a high rate of concordance with the blood culture (p< 0.0001). However, T2C positive cases had blood culture performed in 64% and had lower concordance rate (38.9%) (Table 2). There were 4 cases of candidemia with organisms not detectable by T2C.

Antifungal therapy was administered on 1,232 run days (21.8%) across 182 ECMO runs (45.2%). Median duration of therapy was 4 days [IQR 2-9 days]. The most used antifungal was micafungin (Table 3). There was a statistically significant difference in antifungal exposure with immunocompromised patients receiving longer courses of antifungal therapy (median 6 days vs 4 days, p = 0.0131) and greater number of ECMO days with antifungal therapy (p = 0.0052).

**Conclusion:**

The rate of candidemia in our ECMO cohort was high compared to other high risk populations such as stem cell transplant [4]. Antifungal use was also high and not necessarily pathogen directed, suggesting an opportunity for stewardship or targeted prophylaxis. Finally, our cohort included T2C testing which accounted for 40% of cases. Recognizing the limitations of culture sensitivity, the role for rapid molecular diagnostics in this population warrants further study.

**Disclosures:**

Owen Albin, MD, Biomerieux: Advisor/Consultant|Biomerieux: Grant/Research Support|Charles River Laboratories: Advisor/Consultant Todd P. McCarty, MD, Basilea: Grant/Research Support|Cidara: Grant/Research Support|F2G: Grant/Research Support|Mundipharma: Grant/Research Support|Pfizer: Advisor/Consultant|Scynexis: Grant/Research Support

